# Persistent Transcriptome Alterations in Zebrafish Embryos After Discontinued Opioid Exposure

**DOI:** 10.3390/ijms26104840

**Published:** 2025-05-19

**Authors:** Ryan J. North, Gwendolyn Cooper, Lucas Mears, Brian Bothner, Mensur Dlakić, Christa S. Merzdorf

**Affiliations:** 1 Department of Microbiology and Cell Biology, Montana State University, Bozeman, MT 59717, USA; ryannorth@montana.edu (R.J.N.); lucashmears@gmail.com (L.M.); mdlakic@montana.edu (M.D.); 2 Department of Chemistry and Biochemistry, Montana State University, Bozeman, MT 59717, USA; gwendolyncooper@montana.edu (G.C.); bbothner@montana.edu (B.B.)

**Keywords:** development, zebrafish, opioids, RNA-seq, transcriptomics, ECM disruption, crystallin, low-dose, oxycodone, fentanyl, neurodevelopment, visual development

## Abstract

Much attention has been paid to the public health crisis that has resulted from the opioid epidemic. Given the high number of opioid users that are of childbearing age, the impact of utero exposure is a serious concern. Unfortunately, there is little knowledge regarding the consequences of opioid exposure during early development. While neurobehavioral effects of opioid exposure are well-documented, effects of exposure on embryogenesis remain largely unexplored. To address this gap in knowledge, we investigated the effects of oxycodone and fentanyl exposure on gene expression in zebrafish (*Danio rerio*) embryos using whole embryo RNA sequencing. Embryos were exposed to environmentally relevant (oxycodone HCl 10.6 ng/L and fentanyl citrate 0.629 ng/L) and therapeutically relevant doses (oxycodone HCl 35.14 μg/L and fentanyl citrate 3.14 μg/L) from 2 to 24 h post-fertilization (hpf), followed by another 24 h of opioid-free development. mRNA profiling at 48 hpf revealed dose- and drug-specific gene expression changes. Lower doses of oxycodone and fentanyl both induced more differentially expressed transcripts (DETs) than higher doses, potentially indicative of opioid receptor desensitization occurring at higher concentrations. In total, 892 DETs (corresponding to 866 genes) were identified across all conditions suggesting continued differential gene expression well after cessation of opioid exposure. Gene ontology analysis revealed changes in gene expression relating to extracellular matrix (ECM) organization, cell adhesion, and visual and nervous system formation. Key pathways include those involved in axon guidance, synapse formation, and ECM biosynthesis/remodeling, all of which have potential implications on neural connectivity and sensory development. These findings demonstrate that very early developmental exposure to opioids induces persistent transcriptomic changes which may have lasting implications for vertebrate cellular functions. Overall, these data provide insights into the molecular mechanisms of opioid-induced alterations during development.

## 1. Introduction

Opioids are widely used to treat both chronic and acute pain with roughly 125 million prescriptions dispensed to American patients in 2023 [1]. Though administration of opioids, such as oxycodone, is generally contraindicated in pregnant women, a study of over 1 million pregnant Medicaid recipients in different states reported 9.5–41.6% filling a prescription for opioids while pregnant [2]. The average time of pregnancy awareness is 5.5 weeks, thus avoidance of therapeutic use of opioids would generally not occur during early embryonic development [3]. Currently, the developmental defects caused by gestational opioid exposure are debated; however, numerous studies have noted risks including preterm delivery, reduced fetal growth, congenital abnormalities (including neural tube and CNS defects), and neonatal abstinence syndrome (NAS) [4,5,6]. Associations between opioids and developmental defects or NAS resulted from therapeutic or greater doses. The widespread use of opioids has resulted in environmental presence, with detectable concentrations in wastewater effluent and surface waters [7,8,9]. Given the large potential for exposure and uncertainty regarding the developmental implications, we examined opioid exposure during early development.

Opioid receptors (ORs) belong to the family of G-protein-coupled receptors (GPCRs). They are conserved across vertebrates and mediate the effects of the endogenous opioid system [10]. These receptors include the mu-opioid receptor (MOR, encoded by the *Oprm1* gene), delta-opioid receptor (DOR, encoded by the *Oprd1* gene), kappa-opioid receptor (KOR, encoded by the *Oprk1* gene), and nociceptin/orphanin FQ receptor (NOR, encoded by the *Oprl1* gene) [11,12,13]. Ligand binding activates OR-bound Gi/o G-proteins, where the Ga subunit inhibits adenylyl cyclase and activates G-protein-gated inwardly rectifying potassium (GIRK) channels, leading to reduced cyclic AMP, membrane hyperpolarization, and decreased postsynaptic excitability [13,14]. Concurrent to Ga activation, the GB/y subunit inhibits presynaptic voltage-gated calcium channels, suppressing neurotransmitter release [14,15]. OR activation also engages signaling pathways such as phospholipase C (PLC), MAPK, and PI3K/AKT, regulating gene expression and broad cellular functions [13,16,17,18]. Endogenous OR ligands, including enkephalins, dynorphins, endorphins, and nociceptin/orphanin FQ, are derived from precursor proteins encoded by the *penk*, *pdyn*, *pomc*, and *pnoc* genes, respectively [11]. While ligand-receptor pairing shows some specificity (i.e., enkephalins/DOR, dynorphins/KOR), ligands exhibit overlapping receptor affinities, allowing for functional crosstalk within the opioid system [11].

Exogenous opioids, including natural, semi-synthetic, and fully synthetic compounds, interact with ORs to produce analgesic effects with varying potency. Morphine, codeine, and other naturally-derived opiates were first isolated from *Papaver somniferum* and served as precursors to more potent semi-synthetic and synthetic opioids, such as oxycodone and fentanyl [11,19,20]. Morphine moderately activates MOR (inhibitory constant (Ki), Ki = 1.8 nM) and DOR (Ki = 90 nM) and weakly activates KOR (Ki = 317 nM), influencing stress and dopaminergic pathways [21,22]. Oxycodone engages MOR with a Ki of 18 nM, while more weakly engaging KOR (Ki = 667 nM) and DOR (Ki = 958 nM) [23,24]. Its active metabolite, oxymorphone, is three times more potent than morphine and approximately 12 times more potent than oxycodone, resulting in prolonged activation of opioid receptors (MOR and KOR primarily) [25,26,27,28]. Fentanyl, a highly selective MOR agonist (Ki = 0.135–1 nM), exhibits 50–100 times the potency of morphine due to its rapid and effective receptor activation [29,30,31,32,33].

Danio rerio (zebrafish) are extensively used as model organisms for studying developmental biology and are often employed for toxicology studies due to their high fecundity, external fertilization, ease of visualization, and genetic similarity to humans [34,35]. Zebrafish development during the first 24 h post-fertilization (hpf) parallels the first month of human gestation, encompassing key processes such as gastrulation, neurulation, and segmentation, as well as the initiation of organogenesis [36,37,38].

Zebrafish and humans share conserved genomic and transcriptomic landscapes, including the endogenous opioid system, making zebrafish embryos a powerful model for studying the effects of opioid exposure on developmental processes [34,39]. Maternal transcripts for opioid system components are present during early cleavage stages in both zebrafish and mammals, with opioid ligand transcription beginning early in development [40,41,42]. In zebrafish, *pomca* is expressed by 5.25 hpf, *penka* and *penkb* by 24 hpf, and *pdyn* by 48 hpf, while in mice, ligand expression occurs during early to mid-cleavage stages [43,44].

Opioid/opiate exposure induces significant morphological, cellular, and gene expression changes across developmental stages in both mammals and zebrafish. Morphine and codeine, both naturally occurring opiates, have been studied more extensively for their effects on development than the two common opioids oxycodone (semi-synthetic) and fentanyl (fully synthetic). Studies that exposed zebrafish embryos to 1–10 µM morphine shortly after fertilization until varying times from 24 hpf to 6 days post-fertilization (dpf) observed dose-dependent effects on locomotion and disrupted light/dark behavior, in addition to low mortality and low teratogenic effects [45,46,47,48]. Additionally, embryos allowed to develop to 7 dpf after initial opioid exposure (discontinued at 48 hpf) possessed smaller eyes compared to controls, suggesting that some effects persist beyond the exposure window [48]. For embryos exposed to 10 μM morphine from 5 hpf to 24 or 48 hpf, changes in gene expression implicated pathways related to amino acid and protein metabolism, axonogenesis, neurotransmission, and visual system development [45,46].

Studies on the developmental implications of oxycodone exposure are comparatively sparse. One major study exposed zebrafish embryos to 1.14 μM oxycodone from 24 hpf until analysis at 5 dpf and observed morphology and gene expression changes. The exposed larvae exhibited reduced movement and exploratory behavior, along with differentially expressed genes linked to circadian rhythm, immune regulation, ECM organization, angiogenesis, MAPK activation, and axon guidance [49]. In mammals, oxycodone produces similar effects on immune function, cytoskeletal organization, and axon guidance. One study exposed pregnant rats to approximately 50 μM of oxycodone beginning 15 days prior to mating until weaning and examined offspring brain tissue via RNA sequencing, reporting alterations in genes related to cytokine production, ECM organization, and membrane fusion [50]. A study in mice observed comparable effects [51].

Investigations into developmental fentanyl exposure are more extensive but quite variable. Many fentanyl studies focus on toxicity and lethality which often correspond to respiratory depression (induced in zebrafish with as little as 3 μM fentanyl) [52]. One investigation exposed zebrafish embryos to various fentanyl concentrations (0.01 to 100 μM) from 10 hpf to 8 dpf, finding tail, swim bladder, pericardial, and yolk malformations, along with increased mortality at concentrations above 50 µM [53]. These findings have been corroborated under similar conditions [54]. Another study exposed neuron and glial co-cultures to 0.01 μM or 10 μM fentanyl for 4 days and noted expression changes in genes related to synaptic transmission, inflammation, and ECM organization [55]. Pathways involved in neuronal development and plasticity, ECM organization, and inflammation were also identified in whole larvae (14 dpf) exposed from 2 hpf to 5 dpf [56]. In mammals, fentanyl exposure studies largely center on behavioral outcomes. For example, in mice, self-administration of 32 μM fentanyl during pregnancy increased mortality, reduced litter weight at birth, and produced offspring with behavioral abnormalities, such as depression, anxiety, hyperactivity, and aggression, by 21 days postpartum [57]. Consistent with studies in zebrafish, these mice also displayed sensory deficits.

Morphological and behavioral effects of opioids have been demonstrated to be persistent beyond the exposure window [48,56]. However, whether gene expression alterations persist remains unanswered. To better characterize the effects of exogenous opioids on embryonic development, we employed whole embryo RNA-seq to elucidate transcriptomic alterations after discontinued exposure to either oxycodone or fentanyl at both environmentally and therapeutically relevant doses. To our knowledge, this is the first study utilizing bulk RNA-seq to study the persistent effects of discontinued oxycodone and fentanyl exposure in developing zebrafish embryos.

## 2. Results

### 2.1. Transcriptomic Analysis

**Figure 1:**
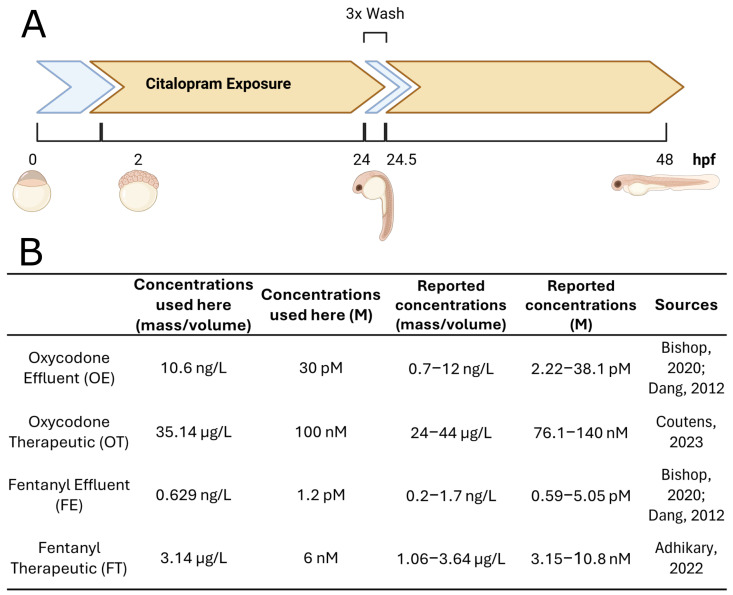
Discontinued exposure protocol and opioid concentrations. (**A**) Discontinued exposure protocol. Exposure of zebrafish embryos to opioids began at approximately 2 hpf and continued through 24 hpf. Embryos were washed 3× with Danieau to remove all residual opioids and allowed to develop for another 24 h in opioid-free Danieau. At 48 hpf, embryos were harvested for RNA extraction. This discontinued exposure protocol was designed to detect lasting transcriptomic changes that persist after cessation of opioid exposure. (**B**) Zebrafish embryos were exposed to the equivalent of either wastewater treatment plant effluent concentrations [9,58] or blood serum concentrations [59,60] resulting from therapeutic doses.

Zebrafish embryos were exposed to opioids from approximately 2 hpf to 24 hpf, followed by 24 h without the presence of opioids and harvest at 48 hpf, which we refer to as the “discontinued exposure protocol” (Figure 1A).

Embryos were exposed to selected oxycodone concentrations (oxycodone effluent, OE; or oxycodone therapeutic, OT) or fentanyl concentrations (fentanyl effluent, FE; or fentanyl therapeutic, FT) (Figure 1B). To assess lasting transcriptomic alterations following discontinued opioid exposure at the described concentrations, we analyzed gene expression in 48 hpf embryos to identify transcriptional changes that persist after drug removal and to shed light on the lasting effects of opioid exposure during very early development.

**Figure 2:**
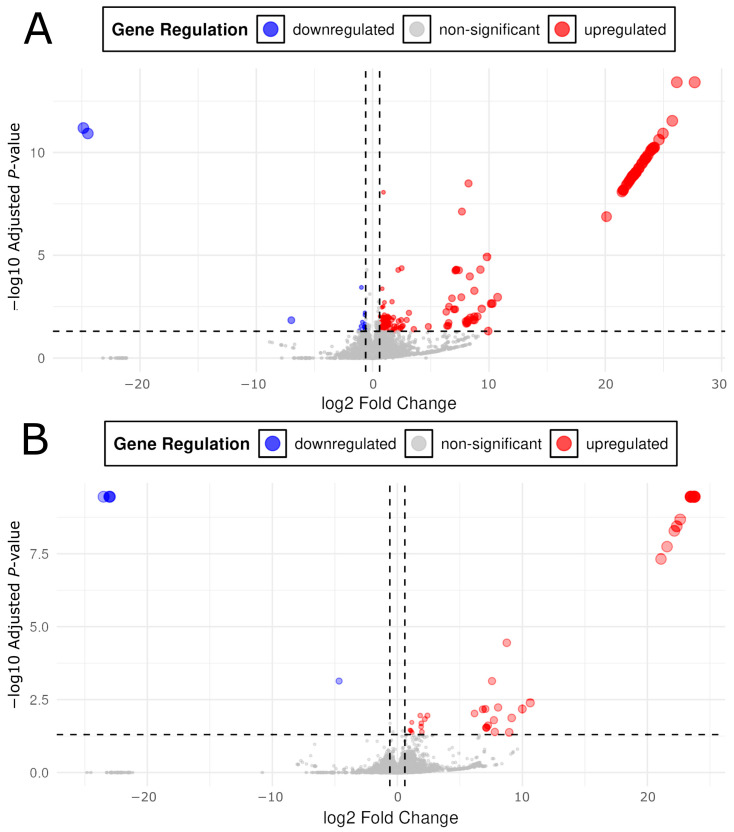
Volcano plots for control vs. oxycodone effluent (**A**) and control vs. oxycodone therapeutic (**B**). *X* axis threshold dotted lines were placed at ±0.6. Data points beyond the 0.6 threshold exhibited an expression change of ±1.5× compared to the control. The *Y* axis threshold dotted line was placed at *p*_adj_ = 0.05. Red colorations are differentially expressed transcripts with a positive log2FC (fold change) and significant *p*_adj_ (≤0.05). Blue coloration is indicative of differentially expressed transcripts with a negative log2FC and significant *p*_adj_ (≤0.05). Due to much smaller *p*_adj_ values in the control vs. OE group, the *Y* axes between (**A**) and (**B**) are differently scaled to accommodate the large −log10 *p*_adj_ values in (**A**).

**Figure 3:**
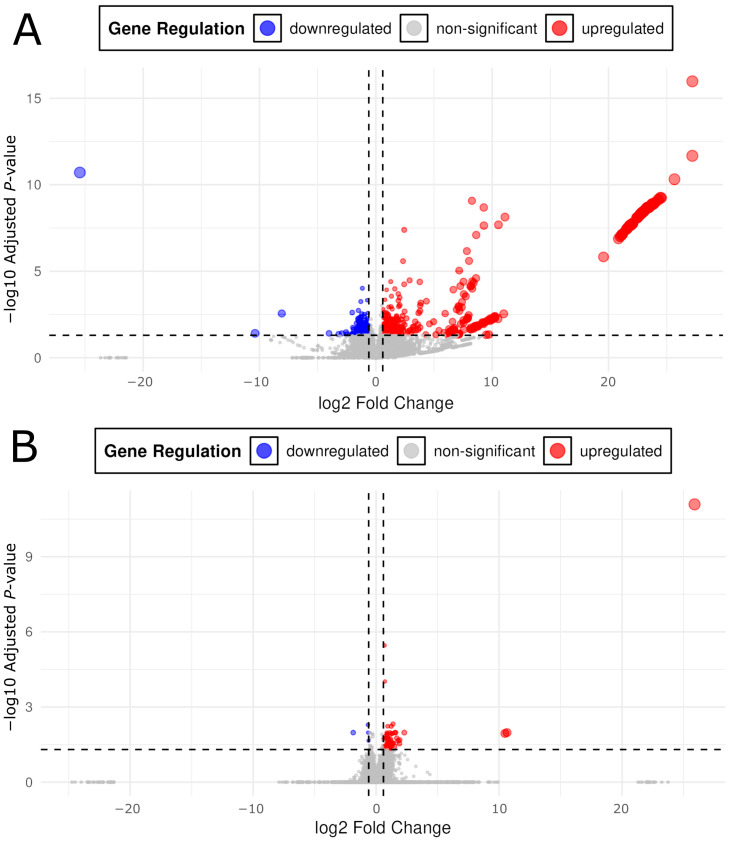
Volcano plots for control vs. fentanyl effluent (**A**) and control vs. fentanyl therapeutic (**B**). *X* axis threshold dotted lines were placed at ±0.6. Data points beyond the 0.6 threshold exhibited an expression change of ±1.5× compared to the control. The *Y* axis threshold dotted line was placed at *p*_adj_ = 0.05. Red colorations are differentially expressed transcripts with a positive log2FC (fold change) and significant *p*_adj_ (≤0.05). Blue coloration is indicative of differentially expressed transcripts with a negative log2FC and significant *p*_adj_ (≤0.05). Due to much smaller *p*_adj_ values in the control vs. OE group, the *Y* axes between (**A**) and (**B**) are differently scaled to accommodate the large −log10 *p*_adj_ values in (**A**).

Over 93% of high-quality reads (Phred ≥ 30) were retained post-QC, with 60–70% of reads successfully mapped to the GRCz11 coding and non-coding RNA reference using Salmon (Table 1A). Differentially expressed transcripts (DETs) were identified based on a threshold of *p*_adj_ ≤ 0.05 and |Log2FC| ≥ 0.6, yielding 892 DETs across all exposure conditions (Table 1B). Exposure-specific transcriptomic responses were observed, highlighting drug- and dose-specific effects. Fentanyl effluent (FE) resulted in 643 DETs (414 upregulated, 229 downregulated), fentanyl therapeutic (FT) yielded 56 DETs (52 upregulated, four downregulated), oxycodone effluent (OE) produced 153 DETs (138 upregulated, 15 downregulated), and oxycodone therapeutic (OT) produced 40 DETs (36 upregulated, four downregulated) relative to control embryos (Table 1B). Upregulated DETs showed larger fold changes than downregulated DETs, with the OT condition exhibiting the largest mean Log2FC for upregulated DETs (10.47, SD = 8.68) and the most extreme downregulated DETs (Log2FC = −18.54, SD = 9.25) (Table 1B). In all cases, opioid exposure resulted in primarily upregulated DETs across a broad range of log2FC values (Figure 2 and Figure 3).

Initial sequencing included N = 5 replicates for each condition. One control replicate failed during sequencing, and another was excluded due to poor quality. To ensure sufficient control replicates, additional N = 5 control samples were sequenced in a separate run. These additional control replicates (R01C, R02C, R03C, R04C, R05C) were combined with three high-quality original controls (R2C, R4C, R5C) to form the final control condition (N = 8). Clustering analysis revealed no evidence of batch effects, as replicates from both sequencing runs displayed similar variance and did not cluster separately. One replicate from both the FE and FT conditions was removed due to quality and outlier analysis parameters. Final sample sizes were as follows: FE (N = 4), FT (N = 4), OE (N = 5), OT (N = 5), and control (N = 8).

### 2.2. Transcriptomic Shifts Are Induced by Opioid Exposure

To investigate transcriptomic variation among exposure groups, principal component analysis (PCA) and partial least squares discriminant analysis (PLS-DA) were performed for each condition (FE, FT, OE, OT) relative to controls (Cs). PCA revealed substantial separation between the control and exposed samples along the first two principal components. PC1 accounted for 21.96%, 28.24%, 25.85%, and 21.7% of the variance in the FE, FT, OE, and OT groups, respectively (Figure S2). PC2 explained 12.26%, 13.86%, 14.45%, and 13.22% of the variance for the respective groups (Figure S2).

**Figure 4:**
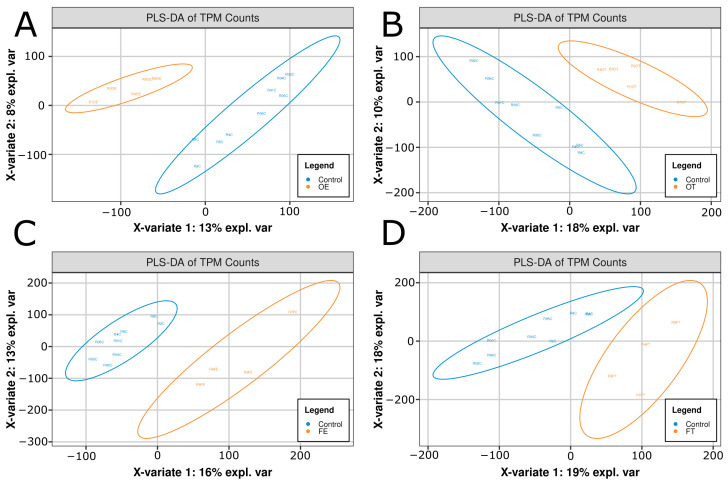
PLS-DA plots comparing control vs. opioid exposure groups. Comparing control (C; N = 8) to (**A**) oxycodone effluent (OE; N = 5), (**B**) oxycodone therapeutic (OT; N = 5), (**C**) fentanyl effluent (FE; N = 4), and (**D**) fentanyl therapeutic (FT; N = 4), resulted in variance-based separation. Total variance (var1 + var2) for OE, OT, FE, and FT were 21%, 28%, 29%, and 37% respectively. Circles indicate 95% confidence intervals of their condition.

PLS-DA confirmed these findings, showing clear separation of control and exposure groups along components 1 and 2 for each condition, with the following percentage of explained variance: FE (16% and 13%, respectively), FT (19% and 18%), OE (13% and 8%), and OT (18% and 10%) (Figure 4). This separation indicates that opioid exposure elicits a distinct transcriptomic shift relative to controls, with clear dose- and drug-specific effects.

### 2.3. Differentially Expressed Genes

**Figure 5:**
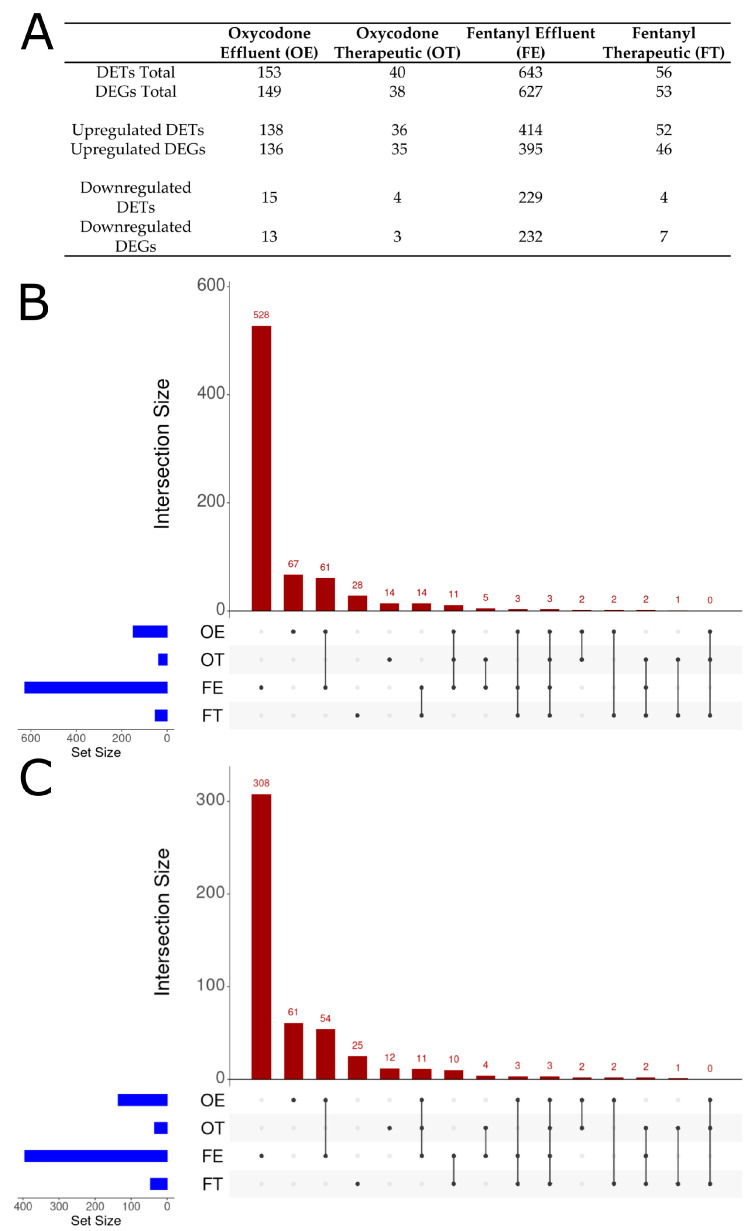
Differentially expressed transcripts (DETs) and genes (DEGs); UpSet plots of DEG control versus opioid exposure conditions. (**A**) Table of DETs and the number of DEGs they represent; reported as total numbers, upregulated subset, and downregulated subset. (**B**) UpSet plot comparing all DEGs for each opioid condition. Horizontal blue lines and their corresponding scale indicate total number of DEGs in the input population. Vertical red bars in the graph are indicative of the number of genes in the samples defined by the dot(s) present below. For example, a single dot in line with FE represents DEGs unique to the FE condition (528 DEGs), whereas dots at OE and FE connected via a line indicate DEGs shared by OE and FE (61 DEGs). Three DEGs were shared by all four conditions. Total pools of DEGs for OE, OT, FE, and FT are 149, 38, 627, and 53, respectively. (**C**) UpSet plot comparing only upregulated DEGs for each opioid condition. Total pools of upregulated DEGs in this plot for OE, OT, FE, and FT are 136, 35, 395, and 46, respectively.

To determine if the embryonic response to opioid exposure was conserved across all exposure concentrations, transcripts were aggregated to their corresponding genes using the g:Profiler g:Convert tool. Due to alternative splicing, some transcripts mapped to the same gene, reducing the total number of differentially expressed genes (DEGs) relative to differentially expressed transcripts (DETs)—except for those of the downregulated FE and FT groups (Figure 5A). This could arise from alternate transcripts experiencing inconsistent directionality in regulation. Frequently, organisms that rely on alternative splicing during development will exhibit intra-gene transcript-level responses that vary in magnitude and direction [61]. As a result, some transcripts may be downregulated while others remain unchanged or upregulated, which may lead to discrepancies between transcript vs. gene-level expression patterns.

UpSet plots were created to illustrate how many DEGs were unique for each exposure group and how many DEGs were shared among exposure groups. UpSet plots were generated for the total identified DEGs (Figure 5B) and for the upregulated DEGs (Figure 5C). The FE condition yielded 627 DEGs, of which 528 (84%) were unique to FE. The OE condition produced 149 DEGs, of which 67 (45%) were unique, while FT yielded 53 DEGs with 28 (55%) unique, and OT produced 38 DEGs with 14 (37%) unique (Figure 5A,B). Shared DEG analysis revealed 61 DEGs uniquely common to FE and OE and not shared by any other condition (54 upregulated, seven downregulated), 14 uniquely shared between FE and FT (10 upregulated, four downregulated), and 11 shared by OT, OE, and FE (all upregulated) (Figure 5B,C). Finally, three DEGs were shared across all four conditions (*crygm2d2*, *crygm2d12*, which are crystallin genes discussed below and *si:dkey-9l20.3*, a predicted adhesion protein). Interestingly, OE and OT share a total of 16 DEGs, FE and FT share a total of 22 DEGs, OE and FE share a total of 78 DEGs, and OT and FT share a total of six DEGs (Figure 5B). This suggests that low concentrations of opioids share more differential expression than any of the other comparisons, including different doses of the same opioid.

### 2.4. Expression of Endogenous Opioid System Genes

Our data indicate that transcript levels of endogenous opioid system genes did not persistently change following any opioid exposure (Figure S3). Transcripts for all components for the canonical endogenous opioid system were detectable (Figure S3). The following opioid receptor genes: opioid growth factor receptor (*ogfr*), opioid growth factor receptor-like 1 (*ogfrl1*), opioid receptor kappa 1 (*oprk1*), opioid receptor delta 1a (*oprd1a*), opioid receptor delta 1b (*oprd1b*), opioid receptor mu 1 (*oprm1*), and opioid growth factor 2 (*ogfr2*) showed no significant expression changes (Figure S3A). Similarly, the following endogenous opioid ligand genes: prepronociceptin a (*pnoca*), proekephalin a (*penka*), prodynorphin (*pdyn*), and proopiomelanocortin (*pomca*) remained unchanged, except for reduced expression of pdyn-202 with both oxycodone effluent (OE) and therapeutic (OT) exposures (Figure S3B). Interestingly, the other *pdyn* transcript (pdyn-201) was unaffected. These results suggest that if alterations to the endogenous opioid system did occur, they were not detected at the transcript level after cessation of exposure.

### 2.5. Pathway Analysis Using Kyoto Encyclopedia of Genes and Genomes/Gene Ontology (KEGG/GO) Terms

To investigate other systems that changed upon exposure to opioids, the functional enrichment analysis of DEGs from each exposure condition relative to the control was conducted using WebGestalt for Gene Ontology (GO) Biological Process (BP) and Kyoto Encyclopedia of Genes and Genomes (KEGG).

For the control vs. OE comparison, KEGG analysis revealed significant enrichment for pyruvate metabolism (FDR < 0.05) (Table S2). Although some GO:BP terms had FDR values > 0.05, several had notable enrichment ratios, including establishment or maintenance of cell polarity, monocarboxylic acid metabolic process, extracellular structure organization, visual system development, and external encapsulating structure organization (Supplemental Excel File). Similar to GO:BP, some KEGG terms also demonstrated low FDRs, but notable enrichment included glycolysis, focal adhesion, and ATP-dependent chromatin remodeling (Supplemental Excel File).

For the control vs. OT comparison, visual system development was the only GO:BP or KEGG term with an FDR < 0.05 (Table S2). However, GO:BP terms with higher FDRs but notable enrichment included sensory perception, cell activation, blood vessel development, and regulation of cell differentiation (Supplemental Excel File).

GO:BP analysis of DEGs from the control vs. FE comparison revealed significant enrichment (FDR < 0.05) for processes related to notochord development, extracellular structure organization, regulation of cell projection organization, cell junction organization, axon development, cell projection morphogenesis, visual system development, and hemopoiesis (Table S2). KEGG pathways enriched in the FE condition included ECM−receptor interaction, focal adhesion, carbon metabolism, the citrate cycle, and virion processes (all with FDR < 0.05) (Table S2).

In the control vs. FT comparison, significantly enriched GO:BP terms (FDR < 0.05) included sensory perception, visual system development, and muscle system processes (Table S2). GO:BP terms with higher FDRs but notable enrichment included calcium ion transport, cellular component assembly involved in morphogenesis, epidermis development, cardiac muscle contraction, and motor protein activity (Supplemental Excel File). The only KEGG pathway with an FDR < 0.05 was cardiac muscle contraction, while higher-FDR but notable pathways included motor proteins, calcium signaling, and the MAPK signaling pathway (Supplemental Excel File).

### 2.6. Clustering Opioid Exposures by Kyoto Encyclopedia of Genes and Genomes/Gene Ontology (KEGG/GO) Terms

To determine if there were pathways shared across opioid exposure groups, functional terms (KEGG, GO: Biological Process (BP), GO: Cellular Component (CC), and GO: Molecular Function (MF)) were separated and organized by the false discovery rate (FDR) and adjusted *p*-values (*p*_adj_). Terms with significant FDR and *p*_adj_ were assigned a value of =2, terms with only significant *p*_adj_ were assigned a value of =1, and all other terms were assigned a value of =0. Opioid exposure conditions (OT, OE, FT, and FE) were then clustered according to hierarchically shared terms in each of the four functional categories. In the functional categories KEGG, GO:BP, and GO:CC, the exposure conditions cluster by concentration rather than opioid type.

**Figure 6:**
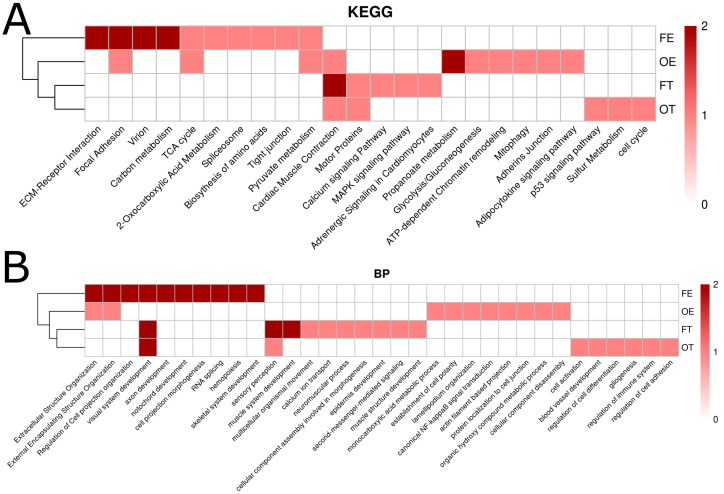
Hierarchical clustering heatmap of KEGG (**A**) and GO:BP (**B**) terms across opioid exposure conditions. Differentially expressed genes (DEGs) from each opioid condition (OE, OT, FE, and FT) compared to the control condition were fed into over-representation analysis tools to derive functional pathway terms from KEGG/GO. Terms with both a significant *p*-value (≤0.05) and false discovery rate (≤0.05; FDR) were ranked as 2. Terms with only a significant *p*-value were ranked as 1. Terms with neither a significant *p*-value or FDR were ranked as 0. Clustering on the left of the heatmaps, produced in pheatmap in R, was carried out to rank similarity across conditions based on shared KEGG/GO term rankings.

There was only a single KEGG common term across three exposure conditions: cardiac muscle contraction (Figure 6A). KEGG terms shared by two conditions include focal adhesion, TCA cycle, pyruvate metabolism, and motor proteins (Figure 6A). Visual system development was the only GO:BP term to be shared by three conditions (Figure 6B). Terms shared by two conditions include extracellular structure organization, external encapsulating structure organization, and sensory perception (Figure 6B).

**Figure 7:**
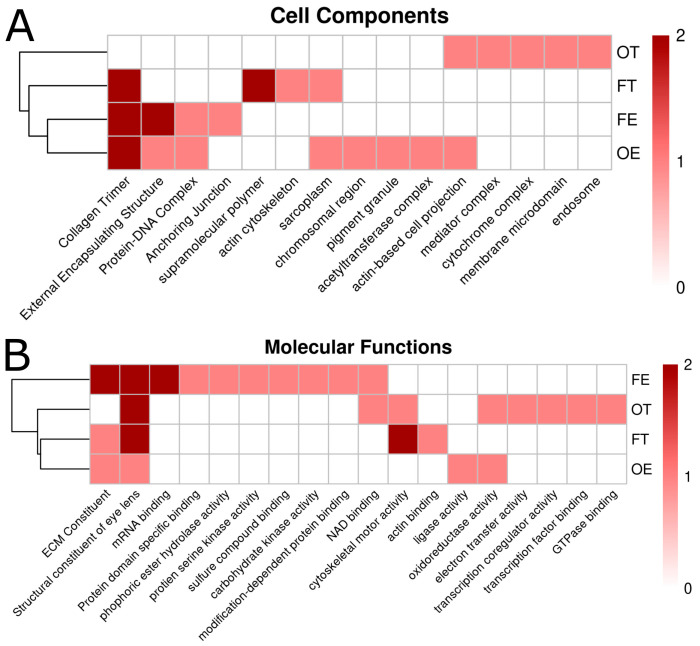
Hierarchical clustering heatmap of Gene Ontology (GO) categories: Cellular Component (CC) (**A**) and Molecular Functions (MFs) (**B**) terms across opioid exposure conditions. Differentially expressed genes (DEGs) from each opioid condition (OE, OT, FE, and FT) compared to the control condition were fed into over-representation analysis tools to derive functional pathway terms from GO. Terms with both a significant *p*-value (≤0.05) and false discovery rate (≤; FDR) were ranked as 2. Terms with only a significant *p*-value were ranked as 1. Terms with neither a significant *p*-value or FDR were ranked as 0. Clustering on the left of the heatmaps, produced via pheatmap in R, was carried out to rank similarity across conditions based on shared GO term rankings.

**Figure 8:**
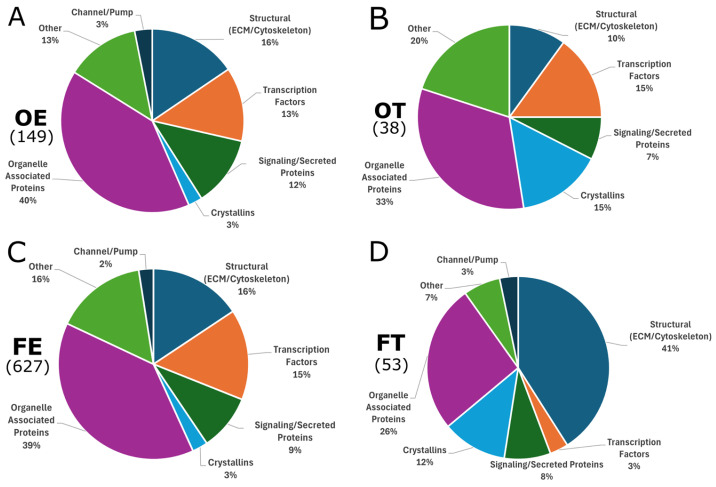
Pie charts of differentially expressed genes (DEGs) from each opioid condition, clustered by functional categories. All DEGs were assigned to one of the following categories: Channel/Pump, Structural (ECM/Cytoskeleton), Transcription Factor, Signaling/Secreted Proteins, Crystallins, Organelle associated proteins, and Other. The (**A**) oxycodone effluent (OE; 149 DEGs) and (**C**) fentanyl effluent (FE; 627 DEGs) charts contained similar percentages of each category. The (**B**) oxycodone therapeutic (OT; 38 DEGs) and (**D**) fentanyl therapeutic (FT; 53 DEGs) charts are less similar. In all conditions, genes coding for Organelle associated proteins or Structural proteins were proportionally most represented.

To build a more complete picture of effects across conditions, we extended our analysis to include both GO:CC and GO:MF. For GO:CC, collagen trimer is the only term shared by three conditions (Figure 7A). Terms shared by two conditions include external encapsulating structure, protein−DNA complex, and actin-based cell projection (Figure 7A). The only term shared by all four opioid exposure conditions is the GO:MF term: structural constituent of eye lens (Figure 7B). ECM constituent is shared by three conditions in this category, as well. Other molecular function terms shared by two conditions include NAD binding, cytoskeletal motor activity, and oxidoreductase activity (Figure 7B). These data suggest that, despite differing opioid exposures, common biological processes and functions are consistently impacted across conditions, alongside distinct, dose-specific effects (Figure 8).

## 3. Discussion

Opioids such as oxycodone and fentanyl are capable of modulating signaling pathways (i.e., PKA, MAPK, NF-κB) via activation of classical opioid receptors [13,17]. These processes are implicated in early developmental processes from fertilization to early organogenesis [37,39]. Morphological effects of opioid exposure during these times have been demonstrated, but the lasting effects on gene expression are poorly established. To determine the effects of opioid exposure on gene expression, we bathed embryos in varying concentrations designed to mimic environmentally and therapeutically relevant amounts of either oxycodone or fentanyl: oxycodone wastewater treatment plant effluent (OE; 10.6 ng/L), oxycodone therapeutic blood serum concentration (OT; 35.14 μg/L), fentanyl effluent (FE; 0.629 ng/L), and fentanyl therapeutic (FT; 3.14 μg/L) (Figure 1B).

These four opioid exposure conditions produced distinct and lasting alterations in embryonic gene expression. Opioid exposure (oxycodone or fentanyl) of zebrafish embryos occurred from approximately 2 hpf to 24 hpf after which embryos continued to develop for another 24 h in opioid-free conditions. Thus, alterations to gene expression at time of harvest (48 hpf) were not due to immediate opioid effects but rather demonstrated lasting changes to the transcriptome. Although opioid conditions showed distinct dose- and drug-specific gene expression profiles, they disrupted common pathways, most notably the expression of genes involved in extracellular matrix structure and visual system development.

Oxycodone treatments produced fewer differentially expressed genes (DEGs) overall compared to the corresponding fentanyl doses (Figure 5A). Interestingly, lower concentrations of oxycodone or fentanyl (wastewater treatment plant effluent concentrations) demonstrated greater numbers of differentially expressed genes than higher doses of oxycodone or fentanyl (blood serum concentrations resulting from therapeutic doses). Specifically, fentanyl effluent led to 643 DETs vs. 56 DETs for fentanyl therapeutic, whereas oxycodone effluent produced 153 DETs vs. 40 DETs for oxycodone therapeutic concentrations (Figure 5A). Additionally, for all opioid conditions, no genes related to the endogenous opioid system (receptors or ligands) showed altered gene expression (Figure S3), suggesting that the observed changes were driven primarily by downstream signaling rather than direct transcriptional changes in the endogenous opioid system.

KEGG/GO pathway analysis highlighted both condition-specific and common impacts of opioid exposure in early developmental stages, with processes related to cell adhesion, visual system development, and focal adhesion shared across groups.

### 3.1. Lower Opioid Doses Resulted in Greater Variety of Differential Expression

The smaller number of DEGs at higher concentrations may be due to opioid desensitization, thus diminishing agonism-based activation of opioid receptors [62]. Desensitization is the process by which the opioid receptor function is attenuated, often via receptor internalization through β-arrestin-mediated endocytosis and subsequent lysosomal degradation [58]. Fentanyl is known induce MOR internalization [59,62]. Some opiates such as morphine can induce desensitization without internalization [60,63], potentially by G-protein decoupling [64]. Oxycodone is among the opioids that do not cause receptor internalization, yet still cause desensitization and tolerance [59,62]. Opioid specificity, dose, and exposure duration govern the rate and extent of desensitization [65,66,67,68,69]. In our study, desensitization may have limited the effects of the therapeutic concentrations on the transcriptome, whereas the lower effluent doses likely sustained receptor signaling, thus producing broader changes in gene expression.

Opioids are known to elicit biphasic, or hormetic, dose-response effects in multiple species and experimental models [70,71,72]. Such effects include alterations in respiratory rate, blood pressure, neutrophil chemotaxis, spontaneous cytotoxicity, and pain [70]. Hormesis in opioid signaling involves differential activation of opioid receptor subtypes and the development of tolerance [70,73]. For example, low ligand concentrations may selectively activate MORs, whereas higher concentrations also engage KORs or DORs, producing distinct downstream outcomes. Similar hormetic patterns have been described for various toxicological agents such as heavy metals, radiation, and alcohol [74,75,76].

Some opioid receptors resist desensitization more effectively than others, depending on the cell type and tissue context [60,77]. Even under high-dose conditions with widespread desensitization, certain receptor populations may continue signaling and produce more localized, targeted effects. This receptor-specific variability adds another layer of complexity to opioid-induced changes in gene expression at different concentrations.

### 3.2. Extracellular Matrix

The extracellular matrix (ECM) is critical for regulating cellular processes, including cell migration, proliferation, differentiation, survival, and communication. These processes are mediated through interactions between ECM structural components (collagen, fibronectin, laminin, etc.) and intracellular cytoskeletal elements (i.e., actin microfilaments) via linker proteins [78,79]. Additionally, enzymes such as Rho GTPases and signaling proteins such as paxillin further coordinate cell–ECM adhesion and signaling [80]. Our data reveal that low-dose opioid receptor activation led to significant changes in ECM-related gene expression, including the genes for various collagens, laminin beta 4, the metallopeptidase ADAMts2, vitrin and periostin (both non-structural ECM proteins), predicted collagen-interacting proteins, and a collagen secretion protein, Creb3l2 (Table 2) [81,82]. Curiously, patterns in ECM gene expression depend on both opioid type and concentration.

Oxycodone therapeutic concentrations produced no change in the expression of collagen genes, while oxycodone effluent concentrations upregulated collagen IV, V, VII, and XXVIII (Tables S1A and S2). Fentanyl therapeutic concentrations strongly upregulated collagen types VII, XVII, and XXIII, whereas fentanyl effluent concentrations impacted collagen types I, II, IV, V, VII, IX, XI, XII, XIV, XVI, XVII, XVIII, XXII, XXIV, XXVII, and XXVIII (Table S1A). Each collagen type fulfills distinct structural and signaling roles. For example, collagens I, II, III, V, XI, XXIV, and XXVII are fibril forming and provide structural integrity to the ECM, whereas the non-fibril forming types of collagen play important roles for ECM organization, adhesion, and cellular signaling [83,84]. Collagen IV (upregulated by OE and FE) is an essential component of basement membranes of epithelial tissues [85,86]. Type V fibrillar collagen (upregulated by OE and FE) is essential for proper ECM formation and functions during development [87]. During wound healing, opioids upregulate many fibrillar collagen genes, including type V, thus altering ECM secretion [88]. The enrichment of ECM secretion pathways often occurs in tandem with inflammatory signaling and dysfunction of ECM remodeling proteins [78], the gene for all of which we observed in the OE and FE. Type VII non-fibrillar collagens (upregulated by OE, FE, and FT) produce anchoring fibrils, connecting the epidermis to the dermis [89]. Such non-fibrillar collagens are important markers of fibrotic deposition, a process often seen with opioid abuse [90]. We see differential expression of some marker genes of fibrosis (i.e., fbn1, tp53inp1, and various NF-κB genes), indicating that fibrotic processes common in opioid abuse [90,91,92] may be present in low-dose exposure during development.

These findings are consistent with previous studies where opioid exposure enhanced collagen production and accumulation as well as bone/cartilage morphogenesis in mice, zebrafish, and cell culture models [79,88,93]. Interestingly, a study demonstrating the effects of fentanyl exposure on neuron and glial co-cultures also reported alterations in ECM-related components (*adamts14*, *col3a1*, *mmp9*), although they observed the downregulation of these ECM genes [55], suggesting that there may be a more nuanced regulatory network for these genes. Our data demonstrated that particularly at ultra-low concentrations, the ECM was affected by opioid exposure. Additionally, fentanyl resulted in more expression changes in collagen-producing genes than did oxycodone (Table S1A), which demonstrates unique effects according to opioid type.

### 3.3. Metalloproteinases

Our data indicate that ECM remodeling persists beyond the cessation of opioid exposure. Fentanyl effluent exposure resulted in upregulated *adamts2*, a metalloproteinase responsible for cleaving fibrillar collagens (I, II, III, and V) [94] 24 h after the exposure window (Table 2). Often, genes responsible for ECM remodeling and ECM production (i.e., metalloproteinases and collagen genes) are expressed with similar directionality during times of largescale ECM alteration [95]. Thus, upregulation of *adamts2* and many collagen genes occurred in parallel (Table S1A). Overall, these observations demonstrate that low-dose opioids can induce continued changes in ECM structure and function, potentially influencing developmental and plasticity-related processes in the nervous system. Furthermore, fentanyl, a strong MOR agonist, exhibited a more profound effect than oxycodone.

Long-term exposure to opioids has been shown to stimulate pro-inflammatory cytokines such as interleukin 33 in brain and spinal cord tissues, which influence ECM remodeling protein activity [78]. These cytokines stimulate NF-κB-signaling in many cell types, including microglia, which drive the expression of genes involved in ECM remodeling that alter astrocyte-neuronal communication, synaptic plasticity, and the trafficking of excitatory receptors [78,96]. This may occur through metalloproteinases, since NF-κB-signaling is known to enhance the expression and activity of both matrix metalloproteinases (MMPs) and ADAMTS proteins [96,97]. Our data show the upregulation of genes for both (Table 2). In addition, we observe the upregulation of genes related to NF-κB-signaling, including the downstream effectors *ror1*, *nlrx1*, and *traf2*, specifically at oxycodone effluent concentrations, although this did not achieve significant false discovery rate (FDR) levels in KEGG/GO analyses. On the other hand, fentanyl effluent exposure elevated *nfkbiaa* and *nfkbib*, which regulate NF-κB-signaling [98,99], indicating a significant link between fentanyl exposure and NF-κB-mediated pathways which have been shown to regulate ECM production and remodeling [78,97,100].

### 3.4. Cell Adhesion

Beyond regulating ECM structural elements, opioids also modulate cell adhesion mechanisms. Cell adhesion occurs via integrin-based interactions with the ECM or through cadherin-mediated cell–cell contacts [101]. Each pathway involves numerous adaptor proteins, signaling mediators, and cytoskeletal regulators. Of these, actin cytoskeletal dynamics and cadherin-based adhesion are emphasized by opioid exposure.

#### 3.4.1. Integrin-Mediated Adhesion and Cytoskeletal Regulation

Integrins are central to cellular connections by bridging ECM and cytoskeleton, usually by relying on intermediary linker proteins. The oxycodone effluent group showed dysregulation of *rac2*, *tncb*, *actb2*, *pak6b*, *pard3ab*, and *tcf7l2*, which serve to regulate cytoskeletal remodeling, cell polarity, as well as ECM binding and organization (Table 2) [102,103,104,105]. Similarly, within the fentanyl effluent group, genes related to focal adhesion and cytoskeletal regulation—*actin b1/2*, *mapk8*, *pak2b*, *prkcba*, *pxna*, *rac2*, *arhgap35b*, and *thbs2b*—were significantly altered compared to controls (Table 2). These genes coordinate integrin signaling and cytoskeletal actin rearrangements required for stable cell–ECM interactions [103,106,107,108]. These findings are consistent with previous studies which have indicated that opioids are able to alter adhesion and cytoskeletal organization via actin regulating GTPases and activation of ERK/MAPK signaling in various models and cell types [109,110,111,112]. At therapeutic concentrations, neither fentanyl nor oxycodone significantly changed adhesion-related genes beyond the collagens mentioned above, indicating that effluent (low-dose) exposures may pose a particular risk for adhesion dysregulation through integrin pathways. Collectively, these changes suggest that embryonic opioid exposure can disrupt integrin-based adhesion, altering cytoskeletal dynamics and focal adhesion integrity.

#### 3.4.2. Cadherin-Mediated Cell–Cell Adhesion

Opioid signaling can also influence cadherin-mediated cell–cell adhesion [80,113]. Our data show that cadherin-related genes were upregulated primarily in fentanyl effluent and, to a lesser degree, oxycodone effluent exposures (Table 2). Specifically, the fentanyl effluent concentration upregulated genes encoding alpha catenin 2 (*ctnna2*), neural cell adhesion molecule 1a (*ncam1a*), and N-cadherin (*cdh2*). The expression of protocadherin 1 gamma (*pcdh1g30*) and FAT atypical cadherin 1a (*fat1a*) was elevated in the oxycodone effluent group, with fat1a also upregulated by fentanyl at therapeutic concentrations (Table 2). No significant cadherin-related gene changes were observed in the oxycodone therapeutic group. These results indicate that opioids differentially modulate cadherin family members, potentially affecting neuronal connectivity, aggregation, and cancer-related processes such as tumor cell detachment and metastasis [113].

The dysregulation of both integrin- and cadherin-mediated adhesion processes suggests broad effects on cytoskeletal arrangement and cell–cell contacts. These findings align with previous studies suggesting that opioid-induced alterations in cell adhesion may have implications for neural development [79,113]. The upregulation of the genes producing laminin beta 4 (a structural component of ECM) and ADAMts2 (a collagen ECM degrading protein) by fentanyl effluent concentrations (Table 2) further highlight how opioids may alter ECM organization, reflecting a nuanced interplay between cell type, opioid receptor subtype, and ECM composition.

### 3.5. Opioids and Visual Development

Visual function depends on proper neural circuitry involving retinal cells and their connections to the brain [114]. Many of the differentially expressed genes affecting visual system development influence axon guidance and synapse formation. For instance, *cyfip2*, *rtn4a*, *pard3ab*, and *smarca4* are central to axon outgrowth and guidance, and *pard3ab* additionally contributes to cell polarity in both neurons and lens fiber cells (Table 2) [115,116,117,118]. Furthermore, genes such as *cyfip2*, *smarca4*, *pard3ab*, *ctbp2a*, *insm1a*, *meis1a*, and *mib1* are key players in retinal development and neuronal morphogenesis (Table 2) [118,119,120,121,122,123]. For example, *meis1a* and *mib1* regulate genes upstream of Notch signaling, which governs differentiation in the lens and neuronal retina, while *cyfip2*, *smarca4*, and *pard3ab* are associated with visual diseases [119,120,121,122,124,125]. The *erg1* and *nr2e3* genes (Table 2) modulate photoreceptor and retinal neuron differentiation [126,127].

Prenatal opioid exposure has been associated with ocular muscle and sensory disorders [33,128]. In zebrafish, much like mammals, the lens is composed of epithelial and fiber cells [129,130,131]. Both cell types contain specialized structural proteins called crystallins [129,130]. Crystallins are water-soluble β-sheet proteins essential for lens transparency, refractive index, and structural integrity; they also have emerging roles in cytoskeletal regulation and apoptosis prevention [129,132]. Two primary crystallin families have been characterized: alpha-crystallins (*crya* genes) and beta/gamma-crystallins (*cryb* and *crygm* genes, respectively). Gamma crystallins are the principal crystallins in both lens epithelial and fiber cells with 37 unique *crygm* genes in zebrafish [129,130]. Our data show that low-dose exposures to oxycodone or fentanyl affected multiple gamma-crystallin genes (Table S1B). The *crygm2d2* and *crygm2d12* genes were upregulated in all four opioid treatment groups (Table S1B). Previous literature has labeled *crygm2d2* and *crygm2d12* as pseudogenes; however, others have noted altered expression of both genes in zebrafish during normal lens cell development and in response to bacterial presence [133,134,135]. Though the explicit functions of these gamma-crystallin 2d subtypes are not clear, we show that their expression was altered by opioid exposure.

Additionally, oxycodone effluent doses altered the expression of four crygm2d genes, while fentanyl effluent doses altered 15 crygm2d genes (Table S1B). The therapeutic doses also induced additional changes in both oxycodone therapeutic altering five crygm2d genes and fentanyl therapeutic altering seven crygm2d genes (Table S1B). Overall, 15 of the 21 crygm2d genes in the zebrafish genome were upregulated by opioid conditions (Table S1B). In addition to gamma-crystallin genes, fentanyl effluent led to changes in crystallin beta gene (*cryba2b*) expression. Previous gene expression studies in both zebrafish and mammals have noted the upregulation of crystallin genes (predominantly alpha-crystallins) in response to opioid exposure [51,136]. In mice, embryonic morphine exposure delayed eye development, reduced lens thickness [137], and codeine caused smaller eyes [48], implying that lens-specific proteins, including crystallins, may be susceptible to opioid-mediated dysregulation. Our findings corroborate these previous studies and indicate that discontinuous exposure of opioids during development alter gamma-crystallin genes and overall visual development pathways.

### 3.6. Axon Guidance and Synapse Formation

A critical component of visual system development is the outgrowth and guidance of retinal ganglion axons (RGAs), which link the retina to the brain [138]. Semaphorins and their receptors, plexins, are important in this process: not only do they guide RGAs, but they also direct endocardial cell and lens epithelial cell migration [139,140]. In addition, rab3 GTPase family members regulate synaptic vesicle trafficking, essential for proper synaptic signaling [141]. We found that zebrafish embryos exposed to fentanyl caused differential expression of plexin B2a (*plxnb2a*) and multiple Rab GTPase pathway components (*rabgap1l*, *rab33a*, *rab11fip1b*, *rab3aa*).

Our analysis identified additional genes associated with both axon growth and visual development, specifically, *gpm6bb*, *sfpq*, *tiam1b*, *vasp*, and *synj1* (Table 2). *synj1* participates in synaptic vesicle trafficking, whereas *GPM6bb* affects membrane trafficking during synapse formation [142,143,144]. *Tiam1b*, *vasp*, and *sfpq* are involved in regulating actin cytoskeleton dynamics necessary for growth cone movement [145,146,147]. The altered expression of genes critical for axon guidance and synaptic function may contribute to subtle yet lasting deficits in neural circuits and visual processing providing a path from opioid exposure to associated behavioral phenotypes.

### 3.7. Extracellular Matrix (ECM), Adhesion, and Visual Development

Opioid-induced perturbations in ECM- and adhesion-related genes described above can further influence lens and neural development. Fentanyl effluent (FE), for instance, significantly upregulated genes involved in cytoskeletal regulation—*plxnb2a*, *sema3*, and *arhgap35b*—each also implicated in axon outgrowth (Table 2) [148,149,150]. *Arhgap35b* modulates Rho GTPase activity, linking changes in cytoskeletal organization with growth cone dynamics [149,151]. Meanwhile, cell adhesion molecules such as *ncam1a*, *cdh2*, and *fat1a*, vital for retinal synapse formation and lens fiber organization [152,153,154,155], were also altered—consistent with the adhesion dysregulation discussed earlier.

Additionally, FE exposure induced changes in ECM components, including collagens, laminins, and integrins, all of which can modulate lens cell formation, elongation, and transparency. Since axon guidance relies on ECM cues, opioids may disrupt both structural and signaling pathways that coordinate proper visual system connections and lens morphology. Genes mediating cytoskeletal changes in axon guidance (*pak2b*, *cyfip2*, *rhoa*) were also upregulated in FE conditions, pointing to a broad, opioid-driven impact on the cytoskeleton across different tissues of the developing visual system. This could have further implications beyond visual development as axon guidance and ECM organization are systemically important throughout all tissues. These transcriptional disruptions align with previous studies that demonstrate impaired neurodevelopment and altered behaviors [45,46,47,48,56,57]. Perturbed regulation of genes involved in ECM organization, adhesion, and cytoskeletal dynamics observed in our study may constitute potential mechanisms for the behavioral and morphological outcomes.

To further illustrate the differences and similarities between opioid exposures, pie charts were created, in which the DEGs of each opioid condition were clustered into discrete categories (Figure 8). As shown in Figure 5, our data identified relatively few individual genes that were shared among the opioid conditions. However, the pie charts representing the DEGs from oxycodone and fentanyl effluent conditions were strikingly similar (Figure 8A,C), indicating their similarity in terms of broader functional categories. Therapeutic conditions of both oxycodone and fentanyl (Figure 8B,D) produced more disparate pie charts. This may be due to the low number of differentially expressed genes for these conditions. Overall, the pie chart for fentanyl therapeutic conditions is most dissimilar compared to the other three pie charts, with the most representative category being structural (ECM/cytoskeleton), thereby representing 41% of DEGs.

## 4. Materials and Methods

### 4.1. Zebrafish Maintenance and Husbandry

Adult *Danio rerio* (zebrafish) were obtained from Carolina Biologicals (Burlington, USA). They were housed and embryos were generated in Montana State University’s Animal Resources Center. All procedures involving animals were conducted in accordance with the guidelines by the Institutional Animal Care and Use Committee (IACUC).

### 4.2. Zebrafish Embryo Treatment and Ribonucleic Acid Extraction

Zebrafish embryos, collected between 0.5 and 1 h post-fertilization (hpf,) were immediately transferred to petri dishes and thoroughly washed with 0.3× Danieau solution [156]. Groups of 60 embryos were exposed to 0.3× Danieau (controls) or either oxycodone HCl (M.W. 351.8 g/mol) or fentanyl citrate (M.W. 528.6 g/mol) in 0.3× Danieau. Fentanyl citrate concentrations included 9.55 nM (=3.14 ng/mL; therapeutic) and 1.91 pM (=0.629 pg/mL; effluent), while oxycodone HCl concentrations were set at 10 nM (=35.14 ng/mL; therapeutic) and 30 pM (=10.6 pg/mL; effluent) (Figure 1B). Effluent group exposure concentrations were derived from reported wastewater plant effluent values [9,157]. The therapeutic concentrations were derived from reported average blood serum levels resulting from therapeutic doses of the respective opioid [158,159]. All embryos exposed to opioid solutions started at approximately 2 hpf and were maintained at 28.5 °C until 24 hpf (approximately 22 h of total exposure). Following exposure, embryos were washed three times with 0.3× Danieau to remove all opioids. They were then returned to 28.5 °C until 48 hpf, at which point they were collected for RNA extraction (Figure 1A). This procedure was repeated with independent clutches of embryos.

The RNA extraction protocol was adapted from Peterson and Freeman [160]. Briefly, 50 embryos were placed into nuclease-free 1.5 mL microcentrifuge tubes, and excess liquid was removed. Each tube received 500 µL of TRIzol reagent (Thermo Fisher Scientific, Cat. No. 15596026, Waltham, USA) and the embryos were homogenized with a P1000 pipette tip, using 40–50 strokes to ensure thorough homogenization. An additional 500 µL of TRIzol was added, followed by vortexing at maximum speed for 1 min. After a 5 min incubation at room temperature, 200 µL chloroform was added to each tube, and the samples were shaken vigorously. The samples underwent a 2 min incubation at room temperature and then were centrifuged at 12,000× *g* for 15 min at 4 °C. The RNA-containing aqueous phase was collected, and RNA was precipitated using isopropanol. The RNA pellet was washed three times with ethanol and resuspended in nuclease-free water. DNase treatment was performed according to the manufacturer’s instructions using DNase I (Thermo Cat. No. EN0521). Subsequently, the RNA samples were purified using the Monarch RNA Cleanup kit (New England Biolabs, Cat. No. T2030L, Ipswich, USA).

RNA integrity was assessed using a Nanodrop spectrophotometer and an Agilent Bioanalyzer 2100 (Agilent RNA 6000 Nano Kit, Cat. No. 5067-1511, Santa Clara, USA). Samples (N = 5) with RNA Integrity Numbers (RINs) ≥ 7 and A260/A280 ratios between 1.8 and 2.0 were selected for further analysis and submitted to the University of Montana Genomics Core (UMGC) for cDNA library preparation (Zymo-Seq RiboFree Total RNA Library Kit (Cat. R30003, Irvine, USA). Paired-end sequencing was conducted using the Illumina NovaSeq X Plus (San Diego, USA) platform, targeting 30 million reads per sample with a read length of 150 bp, resulting in an approximate sequencing depth of 10× coverage. For the control samples, one out of five initially failed during sequencing and another one out of five did not meet QC standards; thus, additional five replicates were prepared asynchronously from the initial experiment and ran separately in an identical fashion to serve as a more complete biological replicate source for the control group (total N = 8).

### 4.3. Software and Computational Pipeline

A general computational pipeline from RNA extraction to differential expression analysis can be found in Figure S1. The raw paired FASTQ files were initially evaluated for quality using FASTQC (version 0.11.9-Java0-11). Following this assessment, the FASTQ files underwent correction and trimming utilizing rCorrector (version 1.0.7) [161] and Trimmomatic (version 0.39) [162]. Trimmomatic was configured to remove adapter sequences with parameters set to 2:30:10:2, along with leading and trailing quality trimming at thresholds of 3 and a minimum read length of 36 bp. Over 93% of reads were retained for all samples post processing. The minimum accepted Phred quality score was set to 30, ensuring high-quality reads for downstream analysis. The processed FASTQ files were then mapped and quantified using Salmon (version 1.10.1) [163], which utilized a reference library constructed from both coding and non-coding RNA sequences from zebrafish, incorporating genomic sequences as decoys. Mapping criteria required that reads be uniquely mapped with concordant alignment of paired sequences to the same transcript, ensuring stringent mapping accuracy and minimizing the influence of multi-mapped reads. These reference sequences were sourced from the GRCz11 assembly of Ensembl [164]. The resulting counts for each sample were aggregated into a master counts matrix, which was subsequently analyzed for differential expression using DESeq2 (within the Trinity RNA-Seq framework, version 2.15.2) [165,166].

### 4.4. Kyoto Encyclopedia of Genes and Genomes/Gene Ontology (KEGG/GO) Pathway Analysis

Differential expression matrices were filtered to identify transcripts with an absolute log2-fold change (log2FC) greater than 0.6 (approximately 1.5 times expression) and an adjusted *p*-value of less than or equal to 0.05. Transcripts meeting both criteria were classified as differentially expressed. Outlier analysis, conducted via principal component analysis (PCA), partial least squares discriminant analysis (PLS-DA), and hierarchical clustering (HC), identified one outlier in the fentanyl effluent (FE) group and one in the fentanyl therapeutic (FT) group, both of which were subsequently removed. All PCA, PLS-DA, and HC analyses were performed using standardized transcript per million (TPM) data. For the KEGG and Gene Ontology (GO) term analysis, the list of differentially expressed transcripts was converted to their corresponding gene IDs using the g:Convert feature of gProfiler (version e111_eg58_f463989d) [167]. This gene list was then submitted to WebGestalt (version 2024) [168] to derive KEGG and GO terms categorized under Biological Process, Cellular Component, and Molecular Function, employing the noRedundant option to ensure non-redundancy. The analyses utilized the Affy zebgene 1.1 st v1 reference set, with redundancy removal configured to Weighted Set Cover and multiple testing correction performed using the Benjamini–Hochberg (BH) method. To corroborate the findings, KEGG and GO terms were also derived through the g:GOSt function of gProfiler (version e111_eg58_f463989d) [167]. GO term tables were compiled using WebGestalt and R. Statistical comparisons, UpSet plots, principal component analyses (PCAs), and partial least squares discriminant analyses (PLS-DAs) were conducted in R version 4.3.2 [169] using the following packages: pheatmap, RColorBrewer, UpSetR, mixOmics, ggplot2, ggrepel, and data.table. Volcano plots and heatmaps were generated utilizing the TrinityRNA pipeline (version 2.15.2) with DESeq2 and R features.

## 5. Conclusions

**Figure 9:**
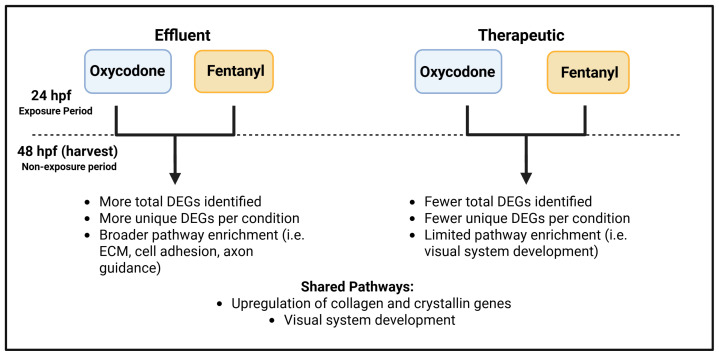
Summary of differential transcriptomic outcomes following discontinued exposure (24 h exposure window followed by 24 h drug-free window), utilizing environmentally relevant (wastewater effluent) or therapeutic concentrations of oxycodone or fentanyl. Effluent exposures (left) induced more total differentially expressed genes (DEGs), more unique DEGs, and broader pathway enrichment in both the oxycodone and fentanyl conditions. Therapeutic concentrations (right) showed fewer gene expression changes and comparably limited pathway enrichment. Shared findings across concentrations included the upregulation of collagen and crystallin genes, all of which are involved in visual system development.

The discontinued oxycodone or fentanyl exposure of zebrafish embryos resulted in distinct changes to gene expression in whole embryo RNA-seq. Opioid exposure occurred during the first 24 hpf which is analogous to the first month of human development. Lower concentrations of both oxycodone and fentanyl resulted in more differential gene expression that persisted for 24 h after cessation of opioid exposure when compared to higher concentrations of both opioids. This difference in DEGs between high vs. low concentrations may be in part due to opioid receptor desensitization caused by the higher opioid concentrations. This deviates from assumptions that higher opioid doses result in greater biological effects, and highlights a more complex relationship between concentration, duration of exposure, and developmental molecular consequences. Given the environmental presence of opioids in wastewater and their potential impact on both human and animal populations, the effects of low-dose embryonic exposures warrant greater attention. Both oxycodone and fentanyl showed alterations in the expression of genes related to extracellular matrix organization, cell adhesion, and visual system development, primarily affecting collagen, cadherin, and crystallin genes. These data demonstrate that opioid exposure, even at extremely low doses, can result in lasting transcriptomic changes that impact crucial processes in embryo development, leading to functional implications (such as behavior and visual sensitization) later in an organism’s life. A summary of major results and conclusions of this work can be found in Figure 9. As all concentrations tested were modeled after doses relevant to human exposure (wastewater treatment plant effluent and clinical therapy), our data suggest long-lasting effects of opioid exposure and potential health concerns from both environmental and clinical exposures.

## Figures and Tables

**Table 1:** Mapping and differentially expressed transcripts.

A	Paired Reads Content Surviving Post-Trim (i)		Total Paired Sequences Post-Trim (ii)	% Successfully Mapped (iii)
Control	92.94%		113,705,405	60.57%
Oxycodone Effluent (OE)	93.69%		109,595,918	66.62%
Oxycodone Therapeutic (OT)	93.86%		113,560,151	65.99%
Fentanyl Effluent (FE)	92.48%		97,358,043	70.8%
Fentanyl Therapeutic (FT)	92.68%		123,727,632	69.24%
**B**	**Oxycodone Effluent (OE)**	**Oxycodone** **Therapeutic (OT)**	**Fentanyl Effluent (FE)**	**Fentanyl Therapeutic (FT)**
Total number of upregulated DETs	138	36	414	52
Average upregulated log2FC	6.75 ± 8.72	10.47 ± 8.68	4.84 ± 6.92	4.37 ± 7.91
Total number of downregulated DETs	15	4	229	4
Average downregulated log2FC	−2.88 ± 7.02	−18.54 ± 9.25	−1.08 ± 1.72	−0.61 ± 0.75
Total number of DETs compared to control	153	40	643	56
Overall average log2FC	5.63 ± 9.05	7.29 ± 12.07	2.6 ± 6.25	4.13 ± 7.77

(**A**) Quality control and mapping percentages. The percentage of paired reads surviving post-trimming and rCorrector protocols were all above 92% (i). All conditions averaged above 90 million paired reads in total post-trimming (ii). Trimmed reads were mapped to a reference library constructed from GRCz11 via Salmon (v1.10.1) with every successful mapping rate above 60% (iii). (**B**) Differentially expressed transcripts across conditions totaled 892 transcripts for opioid vs. control comparisons. For both oxycodone and fentanyl, the lower effluent concentrations produced more DETs than the higher therapeutic concentrations. The average log2-fold change (FC) is a measure of the degree to which gene expression deviates from the control. The average log2FC was generally larger for upregulated transcripts than for downregulated transcripts. Overall, opioid exposure primarily caused the upregulation of gene expression, as reflected by both total number of DETs as well as overall log2FC of DETs.

**Table 2:** Selected differentially expressed genes (DEGs) from significant KEGG/GO pathways based on function and condition.

Gene Name	Description	Condition
ECM-Related		
*col I, II, IV, V VII, IX, XI, XII, XVII, XXVII, XXVIII*	See Table S1A	FE
*col IV, V, VII, XXVIII,*	See Table S1A	OE
*col VII, XVII, XXVIII*	See Table S1A	FT
*adamts2*	disintegrin and metalloproteinase; exercises N-propeptide of fibrillar procollagens type I, II, III, and V	FE
*VIT*	vitrin; ECM protein associated with cell adhesion and migration; expressed highly in the brain	FE
*postn*	periostin; ECM protein associated with regeneration, tissue development, binds to integrins	FE
*si:ch211-196i2.1*	predicted ECM structural protein; predicted involvement in ECM organization	FE
*zgc:113232*	predicted ECM structural protein; predicted involvement in ECM organization	FE
*creb3l2*	cAMP responsive element binding protein 3-like 2; transcriptional activator	FE, OE
*nfkbiaa*	NFKB inhibitor alpha; interacts with REL dimers to inhibit NFKB/REL complex	FE
*nfkbib*	NFKB inhibitor beta; complexes with NFKB and sequesters to cytoplasm	FE
*nlrx1*	NLR family member X1; enhances NFKB kinase dependent signaling	FE, OE
*traf2b*	TNF Receptor Associated Factor; regulates activation of NFKA bad JNK signaling	OE
Cell Adhesion		
*actb*	Actin beta; forms actin filaments essential for cytoskeletal function	FE, OE
*nexn*	Nexilin F-actin binding protein; binds filamentous actin; involved in cell migration and adhesion; important in muscle tissue	OE
*mapk8 and 14*	Mitogen-activated protein kinase; cellular kinase important for proliferation, differentiation, apoptosis	FE
*pak6b*	P21 (RAC1) activated kinase 6; broad cellular function important to cytoskeletal rearrangement, apoptosis, MAPK signaling, and integrin signaling	OE
*pak2b*	P21 (RAC1) activated kinase 2; effector kinase linking Rho GTPases to cytoskeleton reorganization	FE
*prkcba*	Protein kinase C beta a; broad cellular function involved in B cell activation, apoptosis, endothelial cell proliferation, and metabolism	FE
*pxn*	Paxillin; cytoskeletal protein involved in actin-membrane attachment sites of cell adhesion to ECM	FE
*rac2*	Rac family small GTPase 2; GTP metabolizing protein important to secretion, phagocytosis, cell polarization	OE
*thbs2b*	Thrombospondin 2; glycoprotein that mediates cell–cell and cell–ECM interactions	FE
*tncb*	tenascin Cb; acts upstream of axon development, synaptic assembly, collagen ECM regulation, and neuron projection	OE
*pard3ab*	Par-3 Family Cell Polarity regulator alpha; important for asymmetrical cell division, polarized cell growth, tight junction assembly	FE, OE
*tcf7l2*	Transcription factor 7 like 2; key role in Wnt signaling pathway and blood homeostasis	FE, OE
*ctnna2*	Catenin alpha 2; enables actin filament binding and regulation of Arp2/3 complex function; implicated in nervous system function	FE
*ncam1a*	Neural cell adhesion molecule 1; cell adhesion protein involved in cell–cell and cell–ECM interactions; implicated in development of nervous system and nervous system function	FE
*cdh2*	Cadherin 2 (N-type); calcium dependent cell adhesion molecule and glycoprotein; important for left-right axis, nervous system, and cartilage development	FE
*prcdh1g30*	Protocadherin 1; membrane protein found at cell–cell boundaries; involved in neural cell adhesion and development	OE
*fat1a*	FAT atypical cadherin 1; adhesion/signaling molecule implicated in cell proliferation	FE, FT, OE
Lens and Visual Development		
*crygm2d 2, 3, 10, 12, 17*	See Table S1B	OT
*crygm2d 2, 3, 12, 17*	See Table S1B	OE
*crygm2d 1, 2, 5, 9, 12, 14, 16*	See Table S1B	FT
*cryba2b, crygm2d 1, 2, 3, 4, 5, 7, 9, 12, 13, 14, 15, 16, 17, 20*	See Table S1B	FE
*cyfip2*	Cytoplasmic FMR1 interacting protein 2; cell–cell adhesion and post-synapse assembly	FE
*rtn4a*	Reticulon 4; neuroendocrine secretion and membrane trafficking in neuroendocrine cells; neurite outgrowth inhibitor; regeneration of CNS	FE
*smarca4*	SWI/SNF-related BAF chromatin remodeling complex subunit ATPase 4; helicase and ATPase activities implicated in neural stem cell renewal and proliferation as well as broad neuronal function	FE
*insm1a*	INSM transcriptional repressor 1a; neuroendocrine differentiation and promotes neuronal basal progenitor cells	FE
*meis1a*	Meis homeobox 1a; nervous system development and vascular patterning	FE
*mib1*	MIB E3 ubiquitin protein ligase 1; positively regulates NOTCH signaling; important for ubiquitination and apoptosis	FE
Axon Guidance and Synapse Formation		
*plxnb2a*	Plexin B2a; transmembrane receptors important for axon guidance and cell migration; responsive to semaphorins	FE
*rabgap1l*	RAB GTPase activation protein 1-like; enables GTPase activator activity and GTPase binding activity; implicated in cell migration	FE
*rab33a*	RAB33A, member RAS Oncogene Family; small GTPase superfamily that binds GTP and is involved in vesicle transport	FE
*rab3aa*	RAB3A, Member RAS oncogene family; neurotransmitter release cycle, exocytosis, and plasma membrane repair	FE
*gpm6bb*	Glycoprotein M6B; membrane glycoprotein expressed in the brain; involved in membrane trafficking and cell–cell communication	FE
*sfpq*	Splicing Factor Proline and Glutamine Rich; broad cellular functions relating to gene expression; binds histone deacetylases and spliceosome binding	FE
*tiam1*	TIAM Rac1 Associated GEF 1; mediates exchange of GDP for GTP implicated in broad functions including cell shape, migration, adhesion, growth, and polarity	FE
*vasp*	Vasodilator Stimulated Phosphoprotein; implicated in focal adhesion, F-actin formation, cell adhesion, and motility	FE
*synj1*	Synaptojanin 1; regulates levels of membrane phosphatidylinositol-4,5-bisphosphate; implicated in synaptic transmission and membrane trafficking	FE

## Data Availability

Raw fq.gz files for all sequencing data as well as count tables from Salmon processes can be found at NCBI Gene Expression Omnibus under the accession number GSE292183.

## Supplementary Materials

The following supporting information can be downloaded at: https://www.mdpi.com/article/10.3390/ijms26104840/s1.
