# Supplementary material for: Persistent Transcriptome Alterations in Zebrafish Embryos After Discontinued Opioid Exposure
Source: Int J Mol Sci. 2025 May 19;26(10):4840. doi: 10.3390/ijms26104840 (PMC12111994; doi:10.3390/ijms26104840)
Supplement: Supplementary file 1 [file ijms-26-04840-s001.zip › supplemental_figures_and_tables.pdf]

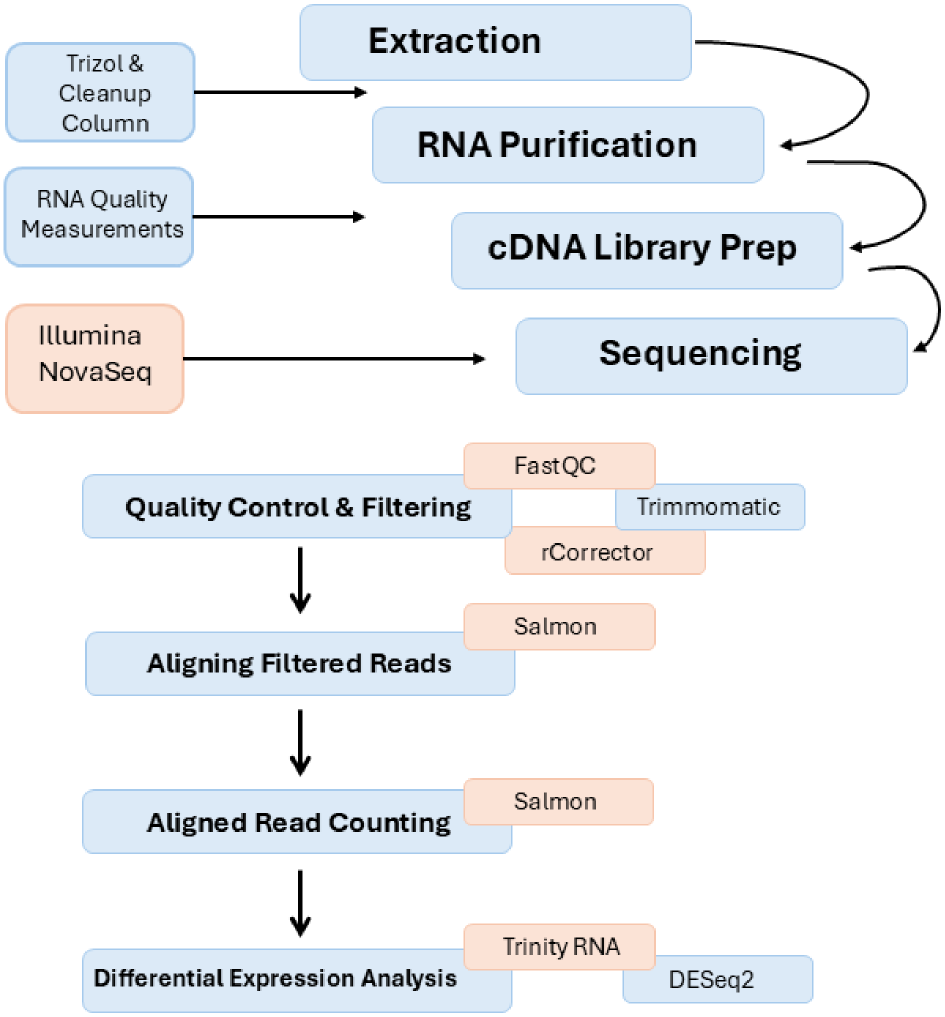

Supplemental Figure S1. RNA sequencing and computational analysis pipeline. Flowchart depicting RNA extraction, sequencing, and computational analysis steps. Total RNA was extracted from 48 hours post-fertilization (hpf) zebrafish embryos using a TRIzol/chloroform protocol adapted from Peterson and Freeman [158], followed by ethanol washes, DNase I treatment, and affinity column purification. RNA quality was assessed using both a NanoDrop spectrophotometer and an Agilent 2100 Bioanalyzer. cDNA synthesis was performed by the University of Montana Genomics Core (UMGC) using Zymo-Seq RiboFree Total RNA Library Kit. Raw sequencing data was obtained from Novogene after sequencing on the Illumina NovaSeq X Plus platform. Subsequent computational analysis included quality trimming with Trimmomatic (v0.39), error correction with rCorrector (v.1.0.7), transcript quantification and mapping with Salmon (v1.10.1), and differential expression analysis using DESeq2 through the TrinityRNA pipeline (v2.15.2).

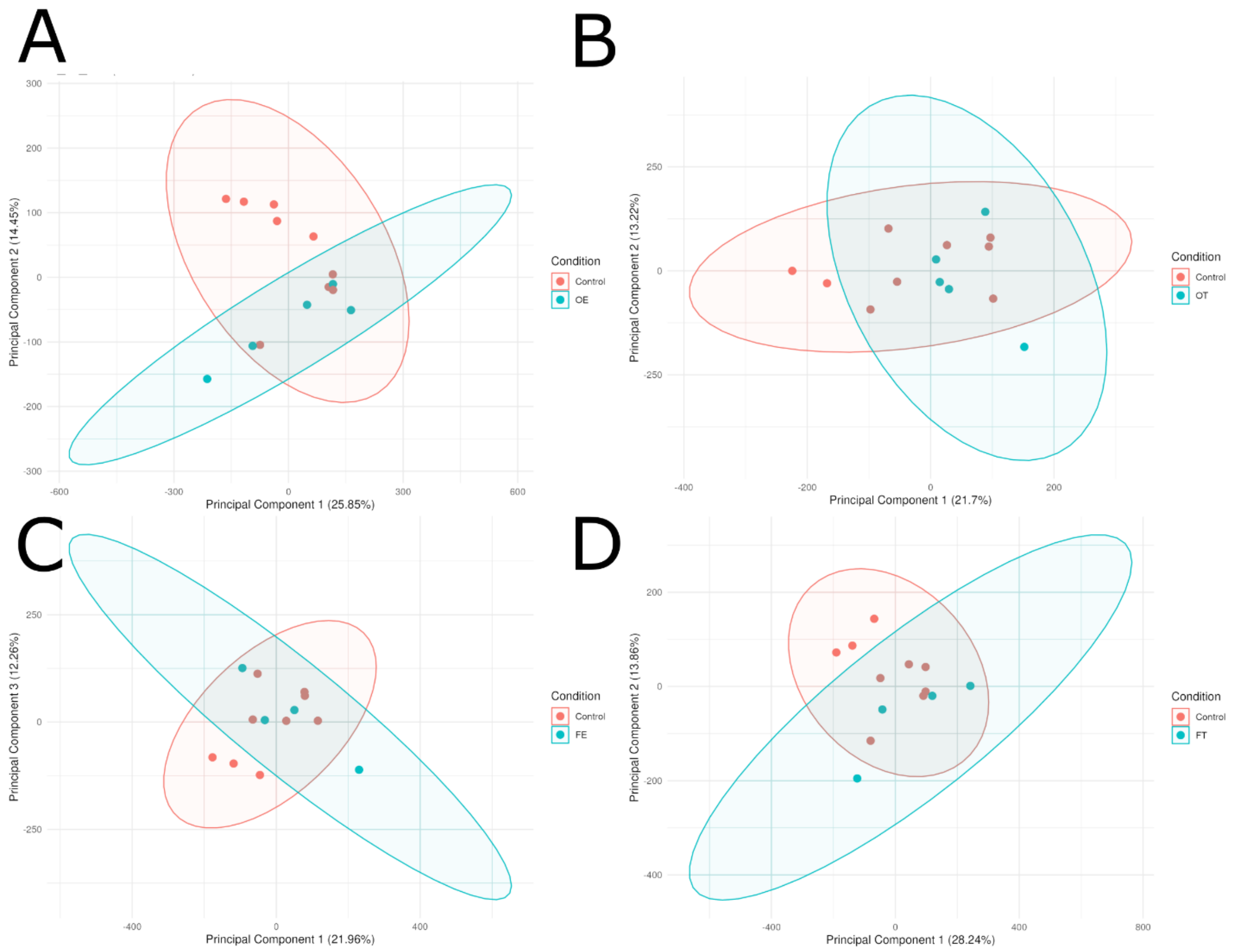

Supplemental Figure S2. Principal component analysis (PCA) plots comparing control vs opioid exposure groups. Controls are compared (C; N=8) to (**A**) Oxycodone effluent (OE; N=5), (**B**) Oxycodone therapeutic (OT; N=5), (**C**) Fentanyl effluent (FE; N=4), and (**D**) Fentanyl therapeutic (FT; N=4) exposure groups, resulted in principal components (PC1 vs PC2) for OE, OT, FE, and FT of 25.85% + 14.45%, 21.7% + 13.22%, 21.96% + 12.26%, and 28.24% + 13.85%, respectively.

A

	Type I			Type II		Type IV			Type V		Type VII	Type IX			Type XI			Type XII	Type XIV	Type XVI	Type XVII	Type XVIII	Type XXII	Type XXIV	Type XXVII	Type XXVIII	
	col1a1a	col1a1b	col1a2	col2a1a	col2a1b	col4a1	col4a2	col4a6	col5a1	col5a3b	col7a1	col9a1b	col9a2	col9a3	col11a1a	col11a1b	col11a2	col12a1a	col14a1b	col16a1	col17a1a	col18a1a	col22a1	col24a1	col27a1b	col28a1a	col28a2a
**OE**	-	-	-	-	-	✓	-	✓	✓	-	✓	-	-	-	-	-	-	-	-	-	-	-	-	-	-	✓	-
**OT**	-	-	-	-	-	-	-	-	-	-	-	-	-	-	-	-	-	-	-	-	-	-	-	-	-	-	-
**FE**	✓	✓	✓	✓	✓	✓	✓	✓	✓	✓	✓	✓	✓	✓	✓	✓	✓	✓	✓	✓	✓	✓	✓	✓	✓	✓	✓
**FT**	-	-	-	-	-	-	-	-	-	-	✓	-	-	-	-	-	-	-	-	-	✓	-	-	-	-	✓	-

B

	**crygm2d1**	**crygm2d2**	**crygm2d3**	**crygm2d4**	**crygm2d5**	**crygm2d7**	**crygm2d9**	**crygm2d10**	**crygm2d12**	**crygm2d13**	**crygm2d14**	**crygm2d15**	**crygm2d16**	**crygm2d17**
**OE**	-	✓	✓	-	-	-	-	-	✓	-	-	-	-	✓
**OT**	-	✓	✓	-	-	-	-	✓	✓	-	-	-	-	✓
**FE**	✓	✓	✓	✓	✓	✓	✓	-	✓	✓	✓	✓	✓	✓
**FT**	✓	✓	-	-	✓		✓	-	✓	-	✓	-	✓	-

Supplemental Table S1. Differential expression of collagen and crystallin genes in response to opioid exposure. (**A**) Differentially expressed collagen genes were detected in all opioid exposure conditions except for OT (Oxycodone therapeutic). FE (Fentanyl effluent) led to widespread differential expression of 27 collagen genes, while FT (Fentanyl therapeutic) affected 3, and OE (Oxycodone effluent) affected 5. These genes span 16 collagen types, each with roles in developmental processes. (**B**) Differentially expressed crystallin genes were observed in all four conditions. FE again showed widespread expression changes, affecting 14 crystallin genes, followed by FT with 7, OT with 5, and OE with 4. The genes shown belong to the crystallin gamma 2d family (crygm2d). Only FE induced differential expression of a non-crygm2d gene, *cryba2a*, part of the beta crystallin family.

Oxycodone Effluent

Gene Set	Description	Size	Expect	Ratio	P Value	FDR
**KEGG**						
dre00620	Pyruvate metabolism	55	0.4438	11.266	7.32E-05	0.012586
**Cellular Component**						
GO:0005581	collagen trimer	57	0.43056	9.2901	0.000851	0.090207

Oxycodone Therapeutic

**Biological Process**						
GO:0150063	visual system development	468	0.76763	7.8162	7.85E-05	0.031001
**Molecular Function**						
GO:0005212	structural constituent of eye lens	63	0.10371	38.568	2.88E-06	0.000536

Fentanyl Effluent

Gene Set	Description	Size	Expect	Ratio	P Value	FDR
**KEGG**						
dre04512	ECM-receptor interaction	93	2.6199	5.3437	2.63E-07	4.52E-05
dre04510	Focal adhesion	258	7.2681	3.1645	8E-07	6.88E-05
dre03265	Virion	8	0.22537	13.312	0.001111	0.047917
dre01200	Carbon metabolism	131	3.6904	2.9807	0.001114	0.047917
**Biological Process**						
GO:0043062	extracellular structure organization	165	4.5493	5.7151	4.88E-13	1.12E-10
GO:0045229	external encapsulating structure organization	166	4.5769	5.6807	5.65E-13	1.12E-10
GO:0031344	regulation of cell projection organization	196	5.404	3.5159	2.02E-06	0.000266
GO:0150063	visual system development	468	12.904	2.4024	5.8E-06	0.000573
GO:0061564	axon development	409	11.277	2.3943	2.51E-05	0.001984
GO:0030903	notochord development	76	2.0954	4.295	0.000233	0.015019
GO:0048858	cell projection morphogenesis	421	11.608	2.1537	0.000266	0.015019
GO:0008380	RNA splicing	284	7.8304	2.4265	0.00034	0.016775
GO:0030097	hemopoiesis	483	13.317	2.0275	0.000398	0.017488
GO:0001501	skeletal system development	341	9.4019	2.2336	0.00051	0.020152
**Cellular Component**						
GO:0005581	collagen trimer	57	1.6146	14.864	5.72E-23	6.07E-21
GO:0030312	external encapsulating structure	292	8.2714	3.5061	2.33E-09	1.23E-07
**Molecular Function**						
GO:0005201	extracellular matrix structural constituent	61	1.6625	13.233	1.49E-19	2.78E-17
GO:0005212	structural constituent of eye lens	63	1.717	8.1536	1.04E-09	9.63E-08
GO:0003729	mRNA binding	214	5.8324	2.7433	0.00025	0.015502

Fentanyl Therapeutic

Gene Set	Description	Size	Expect	Ratio	P Value	FDR
**KEGG**						
dre04260	Cardiac muscle contraction	143	0.3239	15.437	1.16E-05	0.00199
**Biological Process**						
GO:0007600	sensory perception	365	0.68421	10.231	3.29E-06	0.0013
GO:0150063	visual system development	468	0.87729	7.9791	1.68E-05	0.003318
GO:0003012	muscle system process	121	0.22682	17.635	6.97E-05	0.009175
**Cellular Component**						
GO:0099081	supramolecular polymer	470	1.0924	6.408	4.4E-05	0.004666
GO:0005581	collagen trimer	57	0.13248	22.645	0.000279	0.014801
GO:0015629	actin cytoskeleton	261	0.60662	6.5939	0.002562	0.09052
**Molecular Function**						
GO:0005212	structural constituent of eye lens	63	0.15557	44.996	1.21E-10	2.26E-08
GO:0003774	cytoskeletal motor activity	95	0.23459	17.051	8.05E-05	0.007489
GO:0003779	actin binding	317	0.78279	6.3874	0.000948	0.058747

Supplemental Table S2. WebGestalt over-representation analysis (ORA) of KEGG and Gene Ontology (GO) terms for control vs. opioid exposure conditions. Opioid exposure groups include Oxycodone effluent (OE), Oxycodone therapeutic (OT), Fentanyl effluent

(FE), and Fentanyl therapeutic (FT). Functional pathway categories shown are KEGG, GO: Biological Process (BP), GO: Cellular Component (CC), and GO: Molecular Function (MF). For each enriched term, the 'Size' column indicates the total number of genes in the corresponding gene set, 'Expect' represents the expected number of overlapping genes due to chance, and 'Ratio' (Observed/Expected number of genes) denotes the enrichment score. Statistical significance is shown by p-value and false discovery rate (FDR).

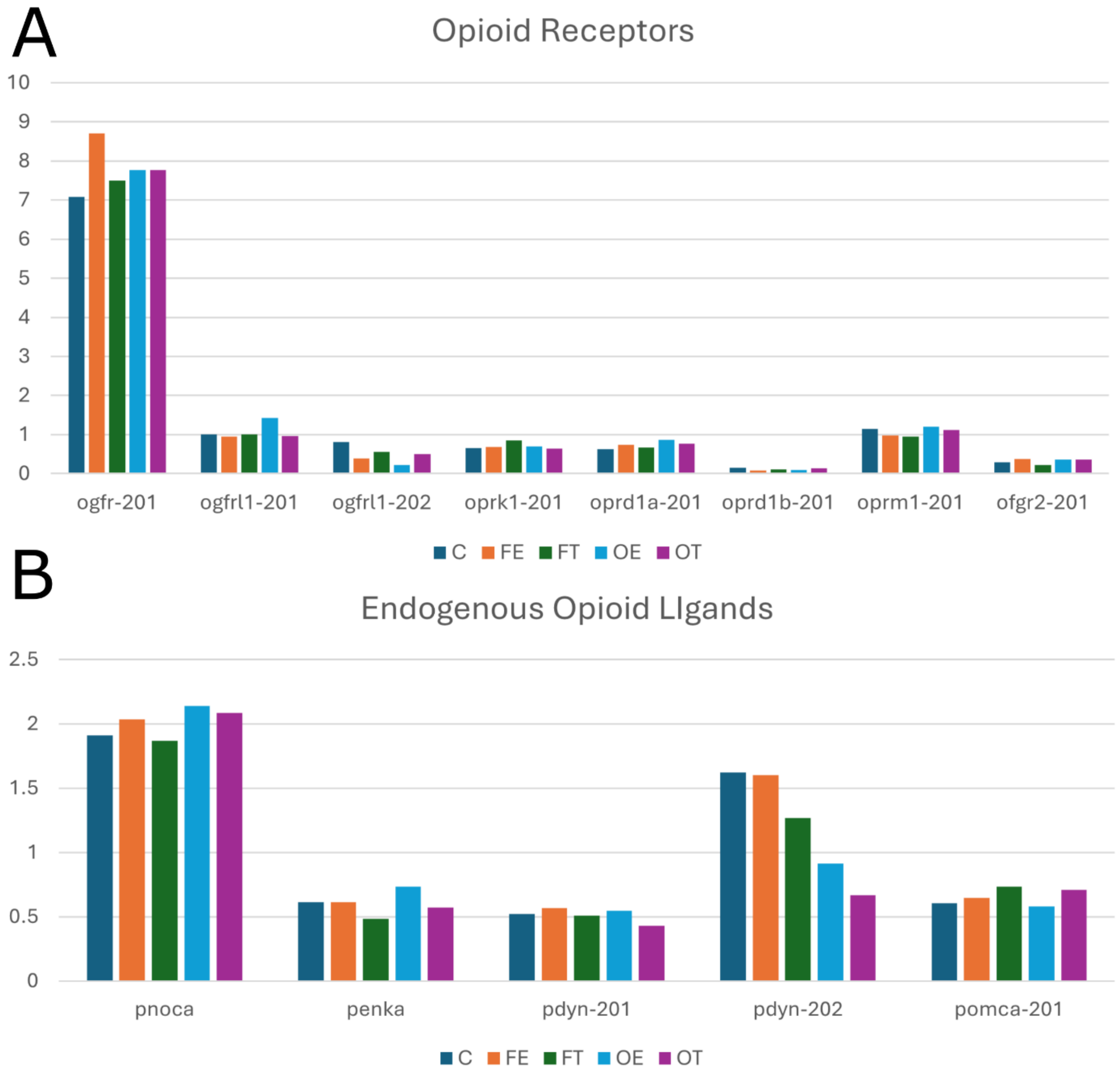

Supplemental Figure S3. Transcript per million (TPM) expression of endogenous opioid system genes. For control and each opioid exposure condition (Oxycodone effluent, OE; Oxycodone therapeutic, OT; Fentanyl effluent, FE; Fentanyl therapeutic, FT) (**A**) TPM values for opioid receptor genes: *ogfr-201*(opioid growth factor receptor), ogfrl1-201/202 (opioid growth factor like 1), oprk1-201 (opioid receptor kappa), oprd1a-201 and oprd1b-201 (opioid growth factors delta 1 and 2), oprm1-201 (opioid receptor mu), and ogfr2-201 (opioid growth factor receptor 2). (**B**) TPM values for endogenous opioid ligand genes: pnoca (prepronociceptin), penka (proenkephalin), pdyn-201/202 (prodynorphin), and pomca-201 (proopiomelanocortin).
